# Intrinsic negative feedback as a limiting factor for the evolution of higher forms of intelligence

**DOI:** 10.12688/f1000research.22039.3

**Published:** 2021-10-19

**Authors:** Stefan T. Arold

**Affiliations:** 1 Division of Biological and Environmental Sciences and Engineering, King Abdullah University of Science and Technology, Thuwal, MK, 23955-6900, Saudi Arabia; 2 Centre de Biologie Structurale, CNRS, INSERM, Université de Montpellier, 34090 Montpellier, France

**Keywords:** iterative evolution, convergent evolution, cognition, complexity, gene expression, sustainability

## Abstract

Longstanding scientific efforts have been dedicated to answer why and how our particular intelligence is generated by our brain but not by the brain of other species. However, surprisingly little effort has been made to ask why no other species ever developed an intelligence similar to ours. Here, I explore this question based on genetic and paleontologic evidence. Contrary to the established view, this review suggests that the developmental hurdles alone are not high enough to explain the uniqueness of human intelligence (HI). As an additional explanation I propose that HI is normally not retained by natural selection, because it is, under most conditions, an intrinsically unfavourable trait. This unfavourableness, however, cannot be explained by physical constraints alone; rather, it may also be rooted in the same emotional and social complexity that is necessary for the development of HI. Thus, a major obstacle towards HI may not be solely the development of the required physical assets, but also to cope with harmful individual, social and environmental feedback intrinsically associated with this trait.

## Introduction

Our particular type of intelligence is commonly believed to be the central feature that has allowed humans to become one of the most abundant mammals on Earth. Yet, although our intelligence is such a powerful and generally useful trait, it has never been paralleled in any other organism. As an explanation, we generally assume that our cognitive uniqueness results from the major difficulty in achieving the required level of developmental sophistication. And after billion years of competitive evolution, we humans were the first species advanced enough to surmount these difficulties and to accumulate all features necessary for achieving this higher intelligence. Here, I investigate this conjecture and its underlying assumptions. For this purpose, I deliberately and necessarily adopt a very narrow anthropocentric definition for human (or human-like) intelligence (HI) as “the intelligence that enables development of advanced technology-based societies like ours”. Within this definition, ‘intelligence’ does not only refer to ‘computing power’, but also to the full ensemble of cognitive and character traits required to reach such a state. This definition is specific for “HI”, formulated to fit the purpose of my manuscript. It should not be seen as an alternative definition for “general intelligence” ^1^.

### A race towards higher intelligence?

First, underlying this conjecture is a worldwide trend toward ever higher levels of intelligence, a trend that reached a critical threshold in the Pliocene when HI was finally developed in humans ^2, 3^. Evidence for such a trend is weak. While a global trend toward bigger brains has been observed in several instances, it does not hold true for all taxonomic levels ^2– 5^. It is unclear if this trend, which is weak and concomitant with an increase in diversity and afflicted by exceptions, is the result of directed evolution or random drift ^4^. It also appears that this trend, if it indeed exists, is a much weaker driving force than are the forces that are linked to adaptation to a specific niche ^6^.

### Linear evolution versus iterative adaptation

**Figure 1.**
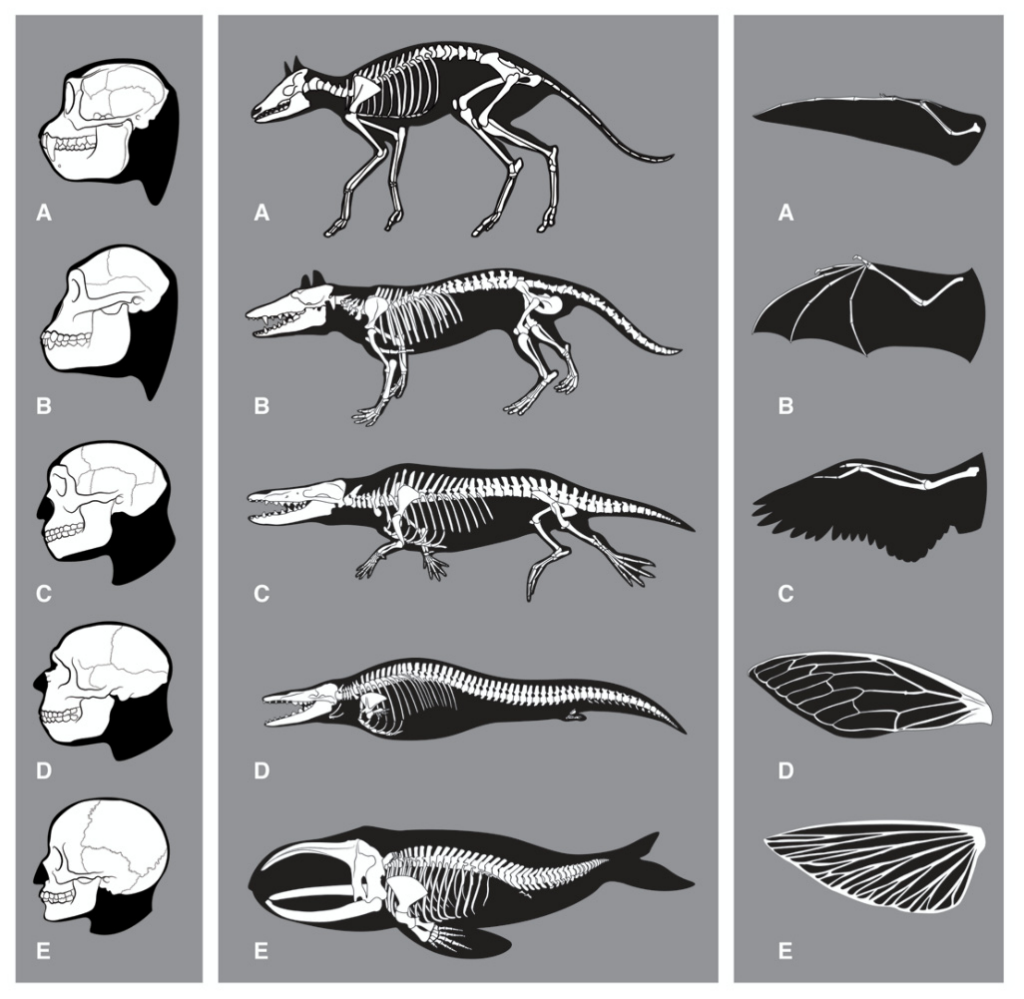
Evolution of features related to thinking, swimming and flying. *Left:* Skeletal changes associated with evolution of the human brain. ( **A**) *Rhinopithecus* (stub-nosed monkey), ( **B**) *Australopithecus*, ( **C**) *Homo erectus*, ( **D**) *Homo neanderthalensis*, ( **E**) *Homo sapiens*. Note that also other anatomical features are likely to be required for human intelligence (HI) (for example those enabling tool-use). *Middle:* Skeletal changes associated with the evolution of whales. Adaptations to the aquatic habitat include: Removing of external appendices (ears, genitals, hind legs); changes of skin; loss of hair; stream-lined body shape; development of a sealable blowhole on top of the head, flippers, tail flukes and a dorsal fin; evolution of a flexible rib cage; increased organ-selective oxygen storage in the body; increased anaerobic capacity of muscles; capacity to slow down pulse ( *bradycdia*); capacity of resting (‘sleeping’) one half of the brain at a time; tail-first birth of babies; development of echolocation ( *odontoceti*). ( **A**) *Diacodexis*, ( **B**) *Pakicetcus*, ( **C**) *Ambulocetus*, ( **D**) *Dorudon*, ( **E**) *Balaena*. Taken from 23. *Right:* Convergent evolution for wing development. ( **A**) pterosaurus, ( **B**) bat, ( **C**) bird, ( **D**) insect, ( **E**) flying fish. Note that although vertebrate forelimbs adaptions are functionally convergent, they are not anatomically convergent. Insect wings are formed from a totally different organ. Although flying and re-adaptation to water are not equivalent to the development of HI on all levels, they might serve as comparison in terms of ‘evolutionary difficulty’ with respect to the required profound reshaping or combination of existing structures (including alterations in bone structure, muscle, metabolism, respiration, vision or connective tissue).

The argument that *Homo sapiens* was the first to reach an HI-enabling level of developmental sophistication evokes a linear view of evolution that posits that basal forms develop into ever more advanced and intelligent organisms. However, paleontologic and genetic evidence shows that evolution is not an orderly step-by-step advancement; rather, it is characterised by consecutive waves of radiation of species (many of which become dead-end groups) into the same ecological niches ^7^. During this iterative process of consecutive adaptations, many sophisticated characteristics (such as vision, flight, echolocation, burrowing or re-adaptation to water) evolved several times, successively and independently, in different species ^7– 12^ ( Figure 1). Even mammalian hallmark features, such as the middle ear and tribosphenic (crushing and biting) molars, evolved not once but several times in different mammals ^7, 13^. This convergence level of evolution indicates that the geological time scale is sufficiently large compared to the biological reproduction and diversification rates that even complex anatomical and molecular features can be reproduced if they enhance the chances of a species’ survival. Since our type of intelligence appears to be such a powerful and versatile development for the survival of a species, why has no other organism acquired HI?

### Lack of suitable environment?

The previous section highlights the importance of ecological niches in directing the evolution of specific traits. Hominid evolution is indeed likely to have been driven by particular changes or variability in climate, although the exact nature and importance of these changes are a matter of debate ^14^. More specifically concerning our cognition, coping with environmental changes has been suggested as a factor that drives brain evolution, within the cognitive buffer hypothesis ^15^. Nonetheless, the climate changes evoked are not sufficiently rare or specific to be a serious limiting factor for the development of HI in other species. Moreover, similar environments, or environmental changes, have failed to promote the emergence of HI in other species. Additionally, if the competitive advantage and driving force of HI development lies in overcoming dependence of the environment ^15^, or enables favourable niche construction ^16^, then this advantage would profit many species, and hence is not a limiting factor. Finally, the rapid and almost worldwide spread of *Homo* species shows that its type of intelligence can be used under many conditions and is not an adaptation limited to a unique environmental ecological niche or geological condition that existed only at the precise time and place of the origin of humans.

### Brain size, structure and complexity

Compared to the brain structure and brain-body ratios of other animals, the human brain is, of course, exceptionally big and complex in terms of layered structure, interconnectedness and neuronal diversity. But is its structure unique and sophisticated enough to justify that our brain capacity was never paralleled through adaptation and convergent evolution? Accumulated evidence suggests that the answer is no. Neuroscientists have established that the brain structures of birds and mammals are never simple. The anatomical differences between species reflect the animals’ adaptations to a particular niche, not the lack of sufficient time for their brains to become more complex ^6^. In fact, the human brain is less evolved (in the sense of being altered with respect to the brains of stem mammalians) than the aye-aye ( *Daubentonia madagascariensis*) brain ^17^. Rather, the human brain is more or less a scaled-up version of a non-human primate brain ^18^. Its size and structure are principally the result of simply extending the high growth rate of foetal brains into early infancy ^19– 22^. This prolonged growth was achieved by the modification of gene expression patterns of a few regulators ^24, 25^, a common mechanism for species differentiation (for example, ^26– 28^). Of course, the scaling-up of the primate brain did not linearly affect all components, and the enhanced increase of the neocortex was certainly key to generating HI. Yet, altering the gene expression pattern of a single transcription factor ( *Pax6*) in mice to a human-like pattern suffices to obtain a primate-like increase in neural progenitors, notably basal radial glia, and ultimately in the number of neurons produced, which is thought to underlie the evolutionary expansion of the neocortex ^25^. Human brain performance was additionally boosted by slightly increasing the ratio of astrocytes to neurons, a tendency already observed in other higher mammals ^29^. This ratio present in humans is, however, similar to that seen in other primates ^18^. In terms of genetic modifications, the development of the human brain therefore appears to be based on extending already existing features through gradual and common mechanisms, rather than being the result of a genetic quantum leap.

Such genetic evolutionary mechanisms are not restricted to primates. For example, it was the expansion of a handful of gene families and genome rearrangements in an otherwise standard invertebrate gene background that allowed octopuses to produce profound morphological and neurological changes ^30^. These changes sufficed to produce a large and sophisticated nervous system in addition to profound morphological innovations for their vision, arms and embryogenesis, resulting in a dramatically increased cognition and behavioural richness in octopuses as compared to worms, molluscs and other lophotrochozoans. Thus, the emergence of our highly specific human cognition has been achieved through continuity on a genetic level, accessible in principle also to other species.

### Convergent evolution of intelligence

Since the human brain originated through extensions of primate brain features, can HI originate only from the brain structure of apes (Hominoidea)? If this is the case, then apes would have had to evolve first before HI occurred, and this condition may help to explain the late and single occurrence of this type of intelligence. However, it was not only other primate species (such as capuchins), but also elephants, cetaceans and certain birds that independently evolved cognitive and social characteristics that are very similar to those of apes (including self-consciousness, grief, altruism, play, envy, compassion and abstract numerical competence), despite having fundamentally different brain architectures ^31– 37^. Although the overall brain architecture differs, convergent evolution can produce obligatory neuronal adaptations in unrelated genera. For example, von Economo neurons, which have been related to complex social behaviour, have independently evolved in primates, whales, elephants and racoons ^38^. Because of neuroplasticity, such convergent evolution is even easier for the brain than for other organs. Accordingly, our particular intelligence is not based on unique characteristics; rather, it arises from a combination and enhancement of abilities found in other vertebrates ^39^. In fact, our large brains and particular intelligence appear to be independent of our ape phylogeny; rather, they result from convergent processes similar to those that produced avian and New World monkey brains ^40^. Taken together with the finding that in some cognitive tasks monkeys can be outperformed by other animals, including birds ^41, 42^ and even fish ^43^, scientific evidence does not support the unilinear view that only the brain structure of apes is suited to produce HI.

### Overcoming the high energy requirement

Big brains are energetically costly organs. Moreover, because of its neuronal density and neuron/glial cell ratio, a human brain requires more energy than for example a rodent brain of the same size ^44^. Could the unique development of HI in humans be explained by our species being the only one capable of coping with the high metabolic demand of a brain required for generating HI?

There are many ways of overcoming metabolic limitations. These include shifting to higher-energy food sources (such as nuts, honey or meat, especially from invertebrates like molluscs or insects); external transformation of food to a more edible state (through grinding, fermentation, cooking or other types of food processing); more efficient use of existing food sources through simple tools and/or strategies (for transport, butchery, or storage); adaptive genetic changes to improve the digestive system (and associated microflora), or to diminish body energy expenditure [such as changes in skin coating (e.g. fur or feathers) or locomotion (e.g. bipedalism, architecture of limbs and feet)]; increasing foraging efficiency through intra-species organisation (e.g. for hunting, gathering, carcass defence or food processing); or shifting to habitats richer in high-protein food sources. A subset of these strategies was sufficient to provide the increased energy demand throughout hominid evolution (see ref. 45 and discussion therein). These strategies (including the cognitive capacities to cook ^46^) do not require features that are only found in humans, making sufficient subsets of them also accessible to other species. Moreover, as the brain of an organism becomes bigger and more powerful, the enhanced cognitive capacities will increasingly enable this organism to implement strategies to enhance energy intake. The cognitive pay-offs of big brains should therefore allow alleviation of the associated energetic cost. In conclusion, while the high energy demand of an HI-capable brain is an important roadblock for the development of HI, the physical and intellectual strategies required to overcome this hurdle are not only accessible to the human body plan. And although the adaptations required to sustain a big brain are profound, they are paralleled, for example, by the metabolic and physical adaptations required for powered flight, which has evolved multiple times in different taxa ( Figure 1).

### Language and intelligence

It appears safe to assume that HI would not be possible without a powerful language. Compared to other animals, the human language appears to distinguish itself in many ways, including the large amount of symbols used (‘vocabulary’), the complex compositional, hierarchical and recursive syntax, and the need to learn the complete language from scratch during an individual’s lifetime ^47^. It has been suggested that language evolution and efficient tool use have stimulated each other mutually ^48^. More generally, it might be that the way of thinking that our language enables is what makes us human ^49, 50^. It is therefore important to discuss whether the uniqueness of HI can be explained by the uniqueness of our language.

Adopting the particular viewpoint of this essay, we then have to ask whether the development of HI-supporting language would be possible for other species. Let us first consider the requirements for producing a sufficient vocabulary. On a purely anatomical or mechanistic level, a HI-supporting language might be any sort of expression not limited to speech (e.g. twitter, tapping, signs, gestures, visual cues) that can produce a sufficient vocabulary space. This requirement does not seem to be restrictive. Moreover, some evidence suggests that the increase in human brain size preceded the development of finely articulated speech ^51^. It was therefore not the pre-existence of an articulate vocalisation, but only a physiological potential for it, that was sufficient for allowing the evolution of HI in humans.

Let us now consider language syntax and acquisition with respect to human uniqueness. It has indeed been proposed that such properties of the human language are neither specific to language nor to humans: Already Darwin had recognized the similarity between birdsong and human speech ^52^. The process and the underlying neuronal circuits by which a young bird learns the songs from an adult ‘tutor’ show indeed strong parallels with speech acquisition in human infants on behavioural, neural and genetic levels ^53, 54^. Moreover, common genetic key players have been identified, including the *FOXP2* gene ^55, 56^. The hierarchical combination of ‘words’ bears resemblance to semantic combinations in primate calls or compass headings in honeybees ^57, 58^. It has therefore been suggested that the human language results from the combination of separate, pre-existing simple systems that may have evolved for other functional tasks ^59^.

In conclusion, while the exact behavioural, neural and molecular links between language, intelligence and behaviour are still emerging and under debate, significant characteristics of human language appear accessible to other species, including non-primates, and are not tied to a specific brain anatomy or size. Thus, current evidence suggests that the capacity for developing a HI-supporting system of communication is not a feature strictly limited to humans or the human brain anatomy or speech production.

### Is there an essential combination of features?

HI poses requirements to a candidate species beyond developing and coping with the appropriate brain anatomy, associated energy demands and language. For example, HI would be most useful in an organism that can use tools and has a complex social system that allows transmitting and accumulating acquired knowledge and culture. Importantly, these individual features synergise with each other (e.g. 48, 60) and may need to evolve as an ensemble. While producing one individual feature may not be particularly difficult, and although combination of pre-existing traits is a common evolutionary mechanism, it could still be that the combined likelihood of assembling all required features in one species is sufficiently small to explain HI’s uniqueness. Unfortunately, it is currently impossible (i) to test which sub-ensemble of characteristics is a strict prerequisite for HI development (as opposed to characteristics which can be developed *en route* towards HI), and (ii) to quantitate the likelihood for the occurrence of such an ensemble in one animal. Thus, we can only approximate this question by asking if the presence of HI-enabling features and capacities was uniquely found in early hominids. In other words, is HI development such a rare event because of a shortage of suitable candidate species?

Tool use is well documented in many animals and is neither restricted to primates (it has been observed in sea otters, elephants and dolphins) nor to mammals (more than 120 cases of tool use in 104 bird species have been reported) ^61– 64^. Many species of dinosaurs, Triassic archosaurs or marsupials were/are bipeds with free, articulate limbs, affording them great potential for tool use ^10^, many relatively small, birdlike dinosaurs, such as *Troodon*, possessed a grasping hand with an opposable digit ^65^, whereas dolphins use tools even though they have no limbs to grasp the tools. Child caring, intra-species communication and even cultural transmissions are documented not only in primates, but also other vertebrates (such as meerkats and fish) and maybe even in insects (e.g., teaching behaviours have been suggested in ants) ^66^. Social complexity as a potential driver for enhanced brain size and social intelligence (the ‘social complexity hypothesis’ ^60^) has been reported in diverse non-human vertebrates (including lemurs, dolphins, parrots and spotted hyenas ^67– 71^, suggesting that social complexity can drive brain evolution in diverse species. With respect to the importance of language, as discussed above, many species possess means of communication which might have the potential to evolve, within a few million years, into a sufficiently complex vocabulary and syntax to synergise with an emerging cognition. Thus, great apes, dolphins and some bird species have reached a high sophistication in terms of vocabulary, semantics, syntax and symbolic references, together with their cognitive capacities ^34, 72, 73^

Finally, development of HI does not appear restricted to organisms with sizes similar to humans. Although the brain-to-body size (encephalization) appears to correlate roughly with cognitive capacity, brains do not have to be above a particular absolute size to produce high levels of intelligence ^74^. For example, despite having a smaller brain than chimpanzees and australopithecines, *Homo floresiensis*, the Indonesian small-bodied ‘Hobbit’ species, was capable of manufacturing tools as advanced as those produced by *Homo* species with a three times larger brain ^75^. Moreover, the complex cognitive abilities of some bird species (including tool use, episodic-like memory, predicting the behaviour of conspecifics based on own experiences and self-recognition) are produced by brains that are less than 10 g in weight ^76^. Thus, HI candidate species are not limited to those animals that have comparable sizes to humans.

In summary, while it is currently impossible to evaluate the evolutionary likelihood for combining the required features into a HI-capable animal, it is clear that many organisms besides humans readily combined several characteristics that could support HI (such as tool use, cultural transmissions, size of groups or body). During development towards HI, these characteristics can in principle be developed further, and completed, as it has been the case in our lineage. And although the combination of features found in early hominids before brain expansion (i.e. free limbs, capacity for grasping, social structure and tool use) are not common, they are not unique either, and it is therefore unlikely that HI was developed only once because no other species would have been a suitable candidate.

### HI as an intrinsically unfavourable trait

According to the findings discussed above, the development of HI is not complex enough to fully explain that it has never been reproduced in any other species than *Homo sapiens*. If HI were within reach of convergent evolution, as my survey suggests, then an intriguing possible explanation for the fact that HI nonetheless evolved only once would be that HI is normally an overall unfavourable feature that is sooner or later sanctioned by selection. Yet, the physical constraints associated with HI appear to be manageable, and the human body is not special enough to suggest that no other organism could have evolved to cope with those constraints. If there is an unfavourable aspect of HI, then it must arise from somewhere other than these physical constraints.

Could it be that some of the same mental and behavioural characteristics that are necessary for the development of HI might become increasingly unfavourable as a species evolves towards HI, creating a negative feedback loop? There is certainly a positive correlation between the proximity of species to HI and the emotional and structural complexity of their individuals and societies (where emotional complexity is defined as having emotional experiences that are broad in range and well differentiated). This correlation is apparent in species with very different brain structures and sizes (cetaceans, elephants, apes, *Cebus* monkeys and some birds ^31, 32, 34, 73, 77^), suggesting that it is an intrinsic hallmark of HI.

Behavioural studies on capuchin monkeys may provide an anecdotal illustration of this concept. These New World monkeys independently evolved an intelligence with certain characteristics similar to that of great apes and humans ^34^. Owing to their particular amalgam of a strong cognitive capacity and an emotional and almost pugnacious character, capuchins have developed tools and strategies that allow them to forage for food that is impossible for most other animals to attain. Their complex social system stimulates learning, the emergence of culture and the establishment of an efficient communal defence against predators ^34^. This type of intelligence, however, produces a society in which individuals spend an excessive amount of time engaged in complex nonproductive or even counterproductive social activities (such as allomothering, non-reproductive sex, apparently non-profit harassment of other animals or harmful “games” such as eyeball poking) and in violent or lethal aggression (the major cause of death for a capuchin monkey is an altercation with another capuchin monkey) ^34^. If a further increase in the monkey’s para-HI intelligence requires increasing the same behavioural and character features that cause counterproductive comportments, then the resulting negative feedback may block development of HI. Conversely, if a candidate species deviates too much from these characteristics, then HI may not result, despite a suitable cognitive power.

In humans, an illustration for the negative effects of HI-associated cognitive complexity is provided by the high incident of cognitive diseases. For example, schizophrenia has been suggested to be ‘the price that *Homo sapiens* pays for language’ ^78^. It has also been shown in animals and humans that the more polymorphic tri-nucleotide repeats are present in the gene *Hdh* (which codes for the protein huntingtin), the higher the capacity of this gene to promote the neural tube formation required for complex brains, but also the higher the probability to develop fatal neurodegenerative Huntington’s disease ^79^. More generally, the genes regulating synaptogenesis and neuronal circuit formation have been associated with an increased risk of mental illnesses ^80, 81^. Based on autism spectrum disorders, it was suggested that brain networks involved in HI-required cognitive skills, such as language and complex social behaviour, have less compensatory mechanisms, and are hence less robust, than more ancient biological functions ^81^. As a final example, the rise of complex diseases (i.e. diseases caused by a combination of genetic, environmental and lifestyle factors) has been linked to the rapid genetic, geographic, dietary and cultural changes associated with HI ^82^. The impact of neurological pathologies increases in high-cognition species, and cumulates in humans, where currently about one in four individuals is affected (source: World Health Organization, 2001). The incidence of mental disorders continues to grow, and with it its individual, social and economic impacts ( World Health Organization, 2019). Accordingly, mental health costs are now the highest single source of global economic burden in the world ^83^. Thus, the increased instability and lability inherently associated with a trajectory towards HI might produce a negative feedback loop counteracting the evolution of HI.

Additionally, there might be a different type of negative feedback loop intrinsic to HI—HI might simply allow a species to become so successful in exploiting food resources that these resources become exhausted. A non-human example is provided by long-tailed macaques living on islands in Thailand. These monkeys developed stone tools that allow foraging on shellfish. Over time, however, this technology is so efficient that the macaques severely deplete the shellfish populations on the islands. This triggers a feedback loop where diminishing prey size results in reduced stone size ^84^. The authors suggest that continuation of this pattern leads to a point where this technology is no longer beneficial or useful to the island macaques, ultimately leading to extinguishing of this technology.

## Conclusion and outlook

A specie’s intelligence is a multidimensional characteristic adapted to maximise the specie’s survival. My analysis suggests that the genetic adaptations required for development of HI might have been within reach of more species than only *Homo sapiens*. However, all other species that have radiated into high-intelligence niches have stagnated at sub-HI levels, often for many million years; only the *Homo* lineage has crossed this barrier in a very short time. The fact that no other species has reached HI appears unsatisfactorily explained by physical constraints of HI. A possible additional explanation may be that the main barrier towards HI is not only the development of the required physical assets, but also that negative feedback from social, behavioural and neuroanatomical complexity, as well as negative environmental feedback make the development of HI increasingly unfavourable.

How could *Homo* overcome this barrier? I speculate that key features may have been that our brain originated very rapidly as an exaptation, not adaptation ^85^, together with an ensemble of favourable anatomical changes (e.g., skeletal proportions, dental function and the respiratory system), in a species with extremely low population densities (typically about 10 individuals per 100 km ^2^ ^86^). This origin may have initially allowed *Homo* to develop the brain structure required for HI, while circumventing or sufficiently attenuating immediate negative feedback associated with the use of HI in a socially complex populous environment where intra-species competition is the biggest threat.

However, as we are becoming overly abundant, and use our brain to its full potential to succeed within our complex societies, the negative side effects become increasingly challenging. Despite the development of psychological, moral, behavioural (‘self-domestication’), pharmacological and technological solutions, we are knowingly and consciously pursuing our unsustainable development that is rapidly destroying the resources upon which we critically depend ^87– 89^. However, globally engaging in the actions required to change our current trajectory and achieve long-term sustainability appears contrary to human nature.

## Data availability

No data are associated with this article.

## References

[1] Sternberg RJ : The augmented theory of successful intelligence.In R. J. Sternberg (Ed.), *Cambridge handbook of intelligence (2nd ed)*. Cambridge University Press.2020;2:679–708. 10.1017/9781108770422.029

[2] Jerison HJ : Brain evolution: new light on old principles. *Science.* 1970;170(3963):1224–1225. 10.1126/science.170.3963.1224 5478198

[3] Kruska DC : On the evolutionary significance of encephalization in some eutherian mammals: effects of adaptive radiation, domestication, and feralization. *Brain Behav Evol.* 2005;65(2):73–108. 10.1159/000082979 15627722

[4] Finarelli JA Flynn JJ : The evolution of encephalization in caniform carnivorans. *Evolution.* 2007;61(7): 1758–1772. 10.1111/j.1558-5646.2007.00131.x 17598754

[5] Montgomery SH Geisler JH McGowen MR : The evolutionary history of cetacean brain and body size. *Evolution.* 2013;67(11): 3339–3353. 10.1111/evo.12197 24152011

[6] Marino L Hof PR : Nature's experiments in brain diversity. *Anat Rec A Discov Mol Cell Evol Biol.* 2005;287(1):997–1000. 10.1002/ar.a.20261 16200645

[7] Luo ZX : Transformation and diversification in early mammal evolution. *Nature.* 2007;450(7172):1011–1019. 10.1038/nature06277 18075580

[8] Varricchio DJ Martin AJ Katsura Y : First trace and body fossil evidence of a burrowing, denning dinosaur. *Proc Biol Sci.* 2007;274(1616): 1361–1368. 10.1098/rspb.2006.0443 17374596 PMC2176205

[9] Nesbitt SJ Norell MA : Extreme convergence in the body plans of an early suchian (Archosauria) and ornithomimid dinosaurs (Theropoda). *Proc Biol Sci.* 2006;273(1590):1045–1048. 10.1098/rspb.2005.3426 16600879 PMC1560254

[10] Brusatte SL Benton MJ Ruta M : Superiority, competition, and opportunism in the evolutionary radiation of dinosaurs. *Science.* 2008;321(5895):1485–1488. 10.1126/science.1161833 18787166

[11] Xu X Zheng X Sullivan C : A bizarre Jurassic maniraptoran theropod with preserved evidence of membranous wings. *Nature.* 2015;521(7550): 70–73. 10.1038/nature14423 25924069

[12] Wang M O'Connor JK Xu X : A new Jurassic scansoriopterygid and the loss of membranous wings in theropod dinosaurs. *Nature.* 2019;569(7755):256–259. 10.1038/s41586-019-1137-z 31068719

[13] Martin T Ruf I : Paleontology. On the mammalian ear. *Science.* 2009;326(5950):243–244. 10.1126/science.1181131 19815765

[14] Gibbons A : Human evolution. How a fickle climate made us human. *Science.* 2013;341(6145):474–479. 10.1126/science.341.6145.474 23908217

[15] Sol D : Revisiting the cognitive buffer hypothesis for the evolution of large brains. *Biol Lett.* 2009;5(1):130–133. 10.1098/rsbl.2008.0621 19049952 PMC2657766

[16] Kendal J Tehrani JJ Odling-Smee J : Human niche construction in interdisciplinary focus. *Philos Trans R Soc Lond B Biol Sci.* 2011;366(1566):785–92. 10.1098/rstb.2010.0306 21320894 PMC3048995

[17] Kaufman JA Ahrens ET Laidlaw DH : Anatomical analysis of an aye-aye brain ( *Daubentonia madagascariensis*, primates: Prosimii) combining histology, structural magnetic resonance imaging, and diffusion-tensor imaging. *Anat Rec A Discov Mol Cell Evol Biol.* 2005;287(1):1026–1037. 10.1002/ar.a.20264 16211637

[18] Azevedo FA Carvalho LR Grinberg LT : Equal numbers of neuronal and nonneuronal cells make the human brain an isometrically scaled-up primate brain. *J Comp Neurol.* 2009;513(5):532–541. 10.1002/cne.21974 19226510

[19] Bush EC Allman JM : The scaling of frontal cortex in primates and carnivores. *Proc Natl Acad Sci U S A.* 2004;101(11):3962–3966. 10.1073/pnas.0305760101 15007170 PMC374352

[20] Leigh SR : Brain growth, life history, and cognition in primate and human evolution. *Am J Primatol.* 2004;62(3):139–164. 10.1002/ajp.20012 15027089

[21] Semendeferi K Lu A Schenker N : Humans and great apes share a large frontal cortex. *Nat Neurosci.* 2002;5(3):272–276. 10.1038/nn814 11850633

[22] Sherwood CC Stimpson CD Raghanti MA : Evolution of increased glia-neuron ratios in the human frontal cortex. *Proc Natl Acad Sci U S A.* 2006;103(37):13606–13611. 10.1073/pnas.0605843103 16938869 PMC1564260

[23] Barton NH Briggs DEG Eisen JA : Evolution. Cold Spring Harbor Laboratory Press.2007. Reference Source

[24] Somel M Liu X Tang L : MicroRNA-driven developmental remodeling in the brain distinguishes humans from other primates. *PLoS Biol.* 2011;9(12):e1001214. 10.1371/journal.pbio.1001214 22162950 PMC3232219

[25] Wong FK Fei JF Mora-Bermúdez F : Sustained Pax6 Expression Generates Primate-like Basal Radial Glia in Developing Mouse Neocortex. *PLoS Biol.* 2015;13(8):e1002217. 10.1371/journal.pbio.1002217 26252244 PMC4529158

[26] Abzhanov A Protas M Grant BR : *Bmp4* and morphological variation of beaks in Darwin's finches. *Science.* 2004;305(5689):1462–1465. 10.1126/science.1098095 15353802

[27] Erickson GM Rogers KC Yerby SA : Dinosaurian growth patterns and rapid avian growth rates. *Nature.* 2001;412(6845):429–433. 10.1038/35086558 11473315

[28] Bhullar BA Marugán-Lobón J Racimo F : Birds have paedomorphic dinosaur skulls. *Nature.* 2012;487(7406):223–226. 10.1038/nature11146 22722850

[29] Nedergaard M Ransom B Goldman SA : *New roles for astrocytes*: redefining the functional architecture of the brain. *Trends Neurosci.* 2003;26(10):523–530. 10.1016/j.tins.2003.08.008 14522144

[30] Albertin CB Simakov O Mitros T : The octopus genome and the evolution of cephalopod neural and morphological novelties. *Nature.* 2015;524(7564):220–224. 10.1038/nature14668 26268193 PMC4795812

[31] Shoshani J Kupsky WJ Marchant GH : Elephant brain. Part I: gross morphology, functions, comparative anatomy, and evolution. *Brain Res Bull.* 2006;70(2):124–157. 10.1016/j.brainresbull.2006.03.016 16782503

[32] Marino L McShea DW Uhen MD : Origin and evolution of large brains in toothed whales. *Anat Rec A Discov Mol Cell Evol Biol.* 2004;281(2):1247–1255. 10.1002/ar.a.20128 15497142

[33] Reiner A Yamamoto K Karten HJ : Organization and evolution of the avian forebrain. *Anat Rec A Discov Mol Cell Evol Biol.* 2005;287(1):1080–1102. 10.1002/ar.a.20253 16206213

[34] Perry S : Manipulative Monkeys. The Capuchins of Lomas Barbudal. Harvard University Press.2011. Reference Source

[35] Scarf D Hayne H Colombo M : Pigeons on par with primates in numerical competence. *Science.* 2011;334(6063):1664. 10.1126/science.1213357 22194568

[36] Schloegl C Schmidt J Boeckle M : Grey parrots use inferential reasoning based on acoustic cues alone. *Proc Biol Sci.* 2012;279(1745):4135–4142. 10.1098/rspb.2012.1292 22874753 PMC3441070

[37] McNally L Brown SP Jackson AL : Cooperation and the evolution of intelligence. *Proc Biol Sci.* 2012;279(1740):3027–3034. 10.1098/rspb.2012.0206 22496188 PMC3385471

[38] Allman JM Tetreault NA Hakeem AY : The von Economo neurons in the frontoinsular and anterior cingulate cortex. *Ann N Y Acad Sci.* 2011;1225:59–71. 10.1111/j.1749-6632.2011.06011.x 21534993 PMC3140770

[39] Roth G Dicke U : Evolution of the brain and intelligence in primates. *Prog Brain Res.* 2012;195:413–430. 10.1016/B978-0-444-53860-4.00020-9 22230639

[40] Lefebvre L : Brains, innovations, tools and cultural transmission in birds, non-human primates, and fossil hominins. *Front Hum Neurosci.* 2013;7:245. 10.3389/fnhum.2013.00245 23761751 PMC3674321

[41] Bolhuis JJ Wynne CD : Can evolution explain how minds work? *Nature.* 2009;458(7240):832–833. 10.1038/458832a 19370014

[42] Emery NJ Clayton NS : The mentality of crows: convergent evolution of intelligence in corvids and apes. *Science.* 2004;306(5703):1903–1907. 10.1126/science.1098410 15591194

[43] Salwiczek LH Prétôt L Demarta L : Adult cleaner wrasse outperform capuchin monkeys, chimpanzees and orang-utans in a complex foraging task derived from cleaner--client reef fish cooperation. *PLoS One.* 2012;7(11):e49068. 10.1371/journal.pone.0049068 23185293 PMC3504063

[44] Fonseca-Azevedo K Herculano-Houzel S : Metabolic constraint imposes tradeoff between body size and number of brain neurons in human evolution. *Proc Natl Acad Sci U S A.* 2012;109(45):18571–18576. 10.1073/pnas.1206390109 23090991 PMC3494886

[45] Wrangham RW Jones JH Laden G : The Raw and the Stolen. Cooking and the Ecology of Human Origins. *Curr Anthropol.* 1999;40(5):567–594. 10.1086/300083 10539941

[46] Warneken F Rosati AG : Cognitive capacities for cooking in chimpanzees. *Proc Biol Sci.* 2015;282(1809):20150229. 10.1098/rspb.2015.0229 26041356 PMC4590439

[47] Hurford JR : Human uniqueness, learned symbols and recursive thought. *Eur Rev.* 2004;12(4):551–565. 10.1017/S106279870400047X

[48] Greenfield PM : Language, Tools, and Brain - the Ontogeny and Phylogeny of Hierarchically Organized Sequential Behavior. *Behav Brain Sci.* 1991;14(4):531–550. 10.1017/S0140525X00071235

[49] Hauser MD Chomsky N Fitch WT : The faculty of language: what is it, who has it, and how did it evolve? *Science.* 2002;298(5598):1569–1579. 10.1126/science.298.5598.1569 12446899

[50] Boeckx C : Biolinguistics: forays into human cognitive biology. *J Anthropol Sci.* 2013;91:63–89. 10.4436/jass.91009 24038628

[51] Capasso L Michetti E D'Anastasio R : A Homo erectus hyoid bone: possible implications for the origin of the human capability for speech. *Coll Antropol.* 2008;32(4):1007–1011. 19149203

[52] Darwin C : The descent of man, and selection in relation to sex. D. Appleton and company, New York. 1871. 10.5962/bhl.title.24784

[53] Bolhuis JJ Gahr M : Neural mechanisms of birdsong memory. *Nat Rev Neurosci.* 2006;7(5):347–357. 10.1038/nrn1904 16760915

[54] Mooney R : Neural mechanisms for learned birdsong. *Learn Mem.* 2009;16(11):655–669. 10.1101/lm.1065209 19850665

[55] Bolhuis JJ Okanoya K Scharff C : Twitter evolution: converging mechanisms in birdsong and human speech. *Nat Rev Neurosci.* 2010;11(11):747–759. 10.1038/nrn2931 20959859

[56] Scharff C Petri J : Evo-devo, deep homology and FoxP2: implications for the evolution of speech and language. *Philos Trans R Soc Lond B Biol Sci.* 2011;366(1574):2124–2140. 10.1098/rstb.2011.0001 21690130 PMC3130369

[57] Arnold K Zuberbühler K : Language evolution: semantic combinations in primate calls. *Nature.* 2006;441(7091):303. 10.1038/441303a 16710411

[58] Riley JR Greggers U Smith AD : The flight paths of honeybees recruited by the waggle dance. *Nature.* 2005;435(7039):205–207. 10.1038/nature03526 15889092

[59] Miyagawa S Berwick RC Okanoya K : The emergence of hierarchical structure in human language. *Front Psychol.* 2013;4:71. 10.3389/fpsyg.2013.00071 23431042 PMC3577014

[60] Dunbar RI : The social Brain hypothesis. *Evolutionary Anthropology: Issues, News and Reviews.* 1998;6(5):178–190. 10.1002/(SICI)1520-6505(1998)6:5<178::AID-EVAN5>3.0.CO;2-8

[61] Lefebvre L Nicolakakis N Boire D : Tools and brains in birds. *Behav.* 2002;139:939–973. 10.1163/156853902320387918

[62] Krützen M Mann J Heithaus MR : Cultural transmission of tool use in bottlenose dolphins. *Proc Natl Acad Sci U S A.* 2005;102(25):8939–8943. 10.1073/pnas.0500232102 15947077 PMC1157020

[63] Anderson JR : Gone fishing: tool use in animals. *Biologist (London).* 2002;49(1):15–18. 11852280

[64] Rutz C Klump BC Komarczyk L : Discovery of species-wide tool use in the Hawaiian crow. *Nature.* 2016;537(7620):403–407. 10.1038/nature19103 27629645

[65] Russell DA Dong Z : A nearly complete skeleton of a new troodontid dinosaur from the Early Cretaceous of the Ordos Basin, Inner Mongolia, People's Republic of China. *Can J Earth Sci.* 1993;30(10):2163–2173. 10.1139/e93-187

[66] Franks NR Richardson T : Teaching in tandem-running ants. *Nature.* 2006;439(7073):153. 10.1038/439153a 16407943

[67] Jolly A : Lemur social behavior and primate intelligence. *Science.* 1966;153(3735):501–506. 10.1126/science.153.3735.501 5938775

[68] Connor RC : Dolphin social intelligence: complex alliance relationships in bottlenose dolphins and a consideration of selective environments for extreme brain size evolution in mammals. *Philos Trans R Soc Lond B Biol Sci.* 2007;362(1480):587–602. 10.1098/rstb.2006.1997 17296597 PMC2346519

[69] Hobson EA Avery ML Wright TF : The socioecology of Monk Parakeets: Insights into parrot social complexity. *The Auk.* 2014;131(4):756–775. 10.1642/AUK-14-14.1

[70] Holekamp KE Dantzer B Stricker G : Brains, brawn and sociality: a hyaena's tale. *Anim Behav.* 2015;103:237–248. 10.1016/j.anbehav.2015.01.023 26160980 PMC4493912

[71] Freeberg TM Dunbar RIM Ord TJ : Social complexity as a proximate and ultimate factor in communicative complexity. *Philos Trans R Soc Lond B Biol Sci.* 2012;367(1597):1785–1801. 10.1098/rstb.2011.0213 22641818 PMC3367695

[72] Marino L : Dolphin cognition. *Curr Biol.* 2004;14(21):R910–911. 10.1016/j.cub.2004.10.010 15530377

[73] Jarvis ED Güntürkün O Bruce L : Avian brains and a new understanding of vertebrate brain evolution. *Nat Rev Neurosci.* 2005;6(2):151–159. 10.1038/nrn1606 15685220 PMC2507884

[74] Willemet R : Reconsidering the evolution of brain, cognition, and behavior in birds and mammals. *Front Psychol.* 2013;4:396. 10.3389/fpsyg.2013.00396 23847570 PMC3696912

[75] Brumm A Aziz F van den Bergh GD : Early stone technology on Flores and its implications for *Homo floresiensis*. *Nature.* 2006;441(7093):624–628. 10.1038/nature04618 16738657

[76] Prior H Schwarz A Gunturkun O : Mirror-induced behavior in the magpie ( *Pica pica*): evidence of self-recognition. *PLoS Biol.* 2008;6(8):e202. 10.1371/journal.pbio.0060202 18715117 PMC2517622

[77] Emery NJ Seed AM von Bayern AM : Cognitive adaptations of social bonding in birds. *Philos Trans R Soc Lond B Biol Sci.* 2007;362(1480):489–505. 10.1098/rstb.2006.1991 17255008 PMC2346513

[78] Crow TJ : Schizophrenia as the price that homo sapiens pays for language: a resolution of the central paradox in the origin of the species. *Brain Res Brain Res Rev.* 2000;31(2–3):118–129. 10.1016/s0165-0173(99)00029-6 10719140

[79] Lo Sardo V Zuccato C Gaudenzi G : An evolutionary recent neuroepithelial cell adhesion function of huntingtin implicates ADAM10-Ncadherin. *Nat Neurosci.* 2012;15(5):713–721. 10.1038/nn.3080 22466506

[80] Yin J Yuan Q : Structural homeostasis in the nervous system: a balancing act for wiring plasticity and stability. *Front Cell Neurosci.* 2014;8:439. 10.3389/fncel.2014.00439 25653587 PMC4299450

[81] Toro R Konyukh M Delorme R : Key role for gene dosage and synaptic homeostasis in autism spectrum disorders. *Trends Genet.* 2010;26(8):363–372. 10.1016/j.tig.2010.05.007 20609491

[82] Gibson G : Decanalization and the origin of complex disease. *Nat Rev Genet.* 2009;10(2):134–140. 10.1038/nrg2502 19119265

[83] Bloom DE Cafiero ET Jané-Llopis E : World Economic Forum. Geneva.2011.

[84] Luncz LV Tan A Haslam M : Resource depletion through primate stone technology. *eLife.* 2017;6: pii: e23647. 10.7554/eLife.23647 28884681 PMC5590808

[85] Gould SJ : Exaptation: A crucial tool for evolutionary psychology. *Journal of Social Issues.* 1991;47(3):43–65. 10.1111/j.1540-4560.1991.tb01822.x

[86] Eriksson A Betti L Friend AD : Late Pleistocene climate change and the global expansion of anatomically modern humans. *Proc Natl Acad Sci U S A.* 2012;109(40):16089–16094. 10.1073/pnas.1209494109 22988099 PMC3479575

[87] Running SW : Approaching the Limits. *Science.* 2013;339(6):1276–1277. 10.1126/science.1235886

[88] Spratt D Dunlop I : Existential climate-related security risk: A scenario approach. breakthroughonline.org.au.2019. Reference Source

[89] IPCC Sixth Assessment Report. Reference Source

