# Supplementary material for: Intrinsic negative feedback as a limiting factor for the evolution of higher forms of intelligence
Source: F1000Res. 2021 Oct 19;9:34. Originally published 2020 Jan 23. [Version 3] doi: 10.12688/f1000research.22039.3 (PMC8408545; doi:10.12688/f1000research.22039.3)
Supplement: Peer review history

# Peer review: Reviewer response for version 2

**DOI:** 10.5256/f1000research.28862.r92656

**Published:** 2021-08-31

**Authors:** Robert J Sternberg

**Affiliations:** 1 Department of Human Development, Cornell University, Ithaca, NY, USA

This interesting article speculates that the reason that human-type intelligence has not evolved in animals other than humans is that, ultimately, human intelligence is evolutionarily maladaptive.  I believe the evidence supports the contention that human-type intelligence has many maladaptive aspects, but I am not convinced that the issue is evolutionary. Moreover, much depends on what one means by “human intelligence.”

 Perhaps the greatest problem with the article as it now stands is the definition of human intelligence. The definition in the article is “the intelligence that enables development of advanced technology-based societies like ours.” This definition of intelligence is nonstandard and appears to be idiosyncratic relatively to standard definitions in the literature on intelligence, which tend to emphasize ability to learn, to adapt to the environment, to reason, and to process information efficiently and effectively (see definitions in Sternberg, 2020a).

 The problem with the author’s definition is that, for most of human history, there was no serious technology, at least not in the sense discussed in the article.  If the goal of intelligence is to enable technology, then it has to be a recent one, not one that is evolutionarily-based. Technology as we know it today is too recent. Yet, the functions of intelligence actually do change over time (Greenfield, 2020) and are somewhat different as a function of culture and place (Sternberg, 2012). If technology is the goal, and so much of technology, as we now know, is destructive, it is no surprise that intelligence could be so dangerous, whether for evolutionary or other reasons.

 The argument that human-type intelligence is maladaptive fits into an existing literature, which is largely not cited.  Consider some examples. First, there are notions of intelligence that are simply different from the conventional one. For example, Sternberg (2019a, 2019b, 2021), in speaking of “adaptive intelligence,” has argued that current human deployment of intelligence has become destructive not only to humans, but also to many other species. It is making the environment unsustainable for human life, a point with which the author apparently agrees. Second, there are broader notions of intelligence that suggest that the conventional view of intelligence is simply too narrow (Gardner, 2011; Sternberg, 2020b)—that there are multiple intelligences that do not fit conveniently into a single notion of general human intelligence. Third, there are theories of different kinds of intelligence that suggest that the conventional notion simply misses important aspects of human functioning, for example, emotional intelligence (e.g., Rivers *et al*., 2020), cultural intelligence (Ang *et al*., 2020), and practical intelligence (e.g., Hedlund, 2020). Fourth, there is the literature on wisdom, suggesting that humans need to apply their intelligence toward some kind of common good in order to thrive and create a sustainable future for themselves (e.g., Grossmann *et al*., 2020; Sternberg & Karami, 2021). Finally, dystopian novels, such as *Brave New World *and *1984, *have shown how human intelligence can lead to catastrophe as well as to success.

 I think there is good evidence that human intelligence can be maladaptive. We scarcely need laboratory experiments when we look at how humans have handled crises such as global climate change, pollution, pandemics, and income disparity.  But are the mechanisms gravitating against human intelligence in animals other than humans evolutionary? That is much less clear.  No other species has, to our knowledge, come close to destroying the environment in the way humans have. There is no evidence that, because they started destroying the environment, evolution of human-like intelligence stopped. Moreover, for an evolutionary mechanism to take hold, the maladaptive trait would likely interfere with reproductive capability, but the people who destroy the Earth are mostly professionals, from politicians to corporate leaders, who are largely past reproductive age.  By the time they succeed in destroying the environment, they are older. Serious mental illness, cited in the article, can interfere with reproduction, but it seems to apply to relatively small proportions of the human population.

 Although I have some disagreements with the author, I agree that “Despite the development of psychological, moral, behavioural (‘self-domestication’), pharmacological and technological solutions, we are knowingly and consciously pursuing our unsustainable development that is rapidly destroying the resources upon which we critically depend. However, globally engaging in the actions required to change our current trajectory and achieve long-term sustainability appears contrary to human nature.”

 Unless intelligence is deployed more adaptively, the world and the creatures in it are at serious risk (Sternberg, 2021). The question is whether we want to create a future for our progeny over many generations, or rather for many generations of viruses, bacteria, and, inevitably, the ever-present cockroaches.

 References

- Ang, S., Ng, K. Y., & Rockstuhl, T. (2020).  Cultural intelligence.  In R. J. Sternberg (Ed.), *Cambridge handbook of intelligence *(2 ^nd^ ed., pp. 780-801).  Cambridge University Press.
- Gardner, H. (2011). *Frames of mind: The theory of multiple intelligences.  *Basic Books.
- Greenfield, P. M. (2020).  Historical evolution of intelligence.  In R. J. Sternberg (Ed.), *Cambridge handbook of intelligence *(pp. 916-939). Cambridge University Press.
- Grossmann, I., Weststrate, N. M., Ardelt, M., Brienza, J. P., Dong, M., Ferrari, M., Fournier, M. A., Hu, C. S., Nusbaum, H. C., & Vervaeke, J. (2020). The science of wisdom in a polarized world: Knowns and unknowns. Psychological Inquiry, 31(2), 103–133. https://doi.org/10.1080/1047840X.2020.1750917
- Hedlund, J. (2020).  Practical intelligence.  In R. J. Sternberg (Ed.), *Cambridge handbook of intelligence *(2 ^nd^ ed., pp. 736-755).  Cambridge University Press.
- Rivers, S. E., Handley-Miner, I. J., Mayer, J. D., & Caruso, D. (2020).  Emotional intelligence.  In R. J. Sternberg (Ed.), *Cambridge handbook of intelligence *(2 ^nd^ ed., pp. 709-735). Cambridge University Press.
- Sternberg, R. J. (2012). Intelligence in its cultural context. In M. Gelfand, C. -Y. Chiu, and Y. -Y. Hong (Eds.), *Advances in cultures and psychology *(Vol. 2, pp. 205-248). New York: Oxford University Press.
- Sternberg, R. J. (2019a).  A theory of adaptive intelligence and its relation to general intelligence. *Journal of Intelligence,* https://doi.org/10.3390/jintelligence7040023
- Sternberg, R. J. (2019b).  Introduction to the *Cambridge Handbook of Wisdom* *: *Race to Samarra: The critical importance of wisdom in the world today.  In R. J. Sternberg & J. Glueck (Eds.), *Cambridge handbook of wisdom *(pp. 3-9).  Cambridge University Press.
- Sternberg, R. J. (2020a).  The augmented theory of successful intelligence.  In R. J. Sternberg (Ed.), *Cambridge handbook of intelligence *(2nd ed., Vol. 2, pp. 679-708).  Cambridge University Press.
- Sternberg, R. J. (Ed.) (2020b). *Cambridge handbook of intelligence *(2 ^nd^ ed.).  New York: Cambridge University Press.
- Sternberg, R. J. (2021). *Adaptive intelligence: Surviving and thriving in a world of uncertainty.  *New York: Cambridge University Press.
- Sternberg, R. J., & Karami, S. (2021).  What is wisdom? A unified 6P framework. *Review of General Psychology,* *https://doi.org/10.1177/1089268020985509* *.  *Online Version.

Is the topic of the opinion article discussed accurately in the context of the current literature?

No

Are arguments sufficiently supported by evidence from the published literature?

Partly

Are all factual statements correct and adequately supported by citations?

Partly

Are the conclusions drawn balanced and justified on the basis of the presented arguments?

Partly

Reviewer Expertise:

Intelligence

I confirm that I have read this submission and believe that I have an appropriate level of expertise to confirm that it is of an acceptable scientific standard.

## References

[1] : Cultural intelligence. In R. J. Sternberg (Ed.), Cambridge handbook of intelligence (2nd ed.). *Cambridge University Press* .2020;780-801

[2] : Frames of mind: The theory of multiple intelligences. *Basic Books* .2011;

[3] : Historical evolution of intelligence. In R. J. Sternberg (Ed.), Cambridge handbook of intelligence. *Cambridge University Press* .2020;916-939

[4] : The Science of Wisdom in a Polarized World: Knowns and Unknowns. *Psychological Inquiry* .2020;31(2) : 10.1080/1047840X.2020.1750917 103-133 10.1080/1047840X.2020.1750917

[5] : Practical intelligence. In R. J. Sternberg (Ed.), Cambridge handbook of intelligence (2nd ed.). *Cambridge University Press* .2020;736-755

[6] : Emotional intelligence. In R. J. Sternberg (Ed.), Cambridge handbook of intelligence (2nd ed.). *Cambridge University Press* .709-735

[7] : Intelligence in its cultural context. In M. Gelfand, C. -Y. Chiu, and Y. -Y. Hong (Eds.), Advances in cultures and psychology (Vol. 2). *Oxford University Press* .2012;205-248

[8] : A Theory of Adaptive Intelligence and Its Relation to General Intelligence. *J Intell* .2019;7(4) : 10.3390/jintelligence7040023 10.3390/jintelligence7040023 31581505PMC6963795

[9] : Introduction to the Cambridge Handbook of Wisdom: Race to Samarra: The critical importance of wisdom in the world today. In R. J. Sternberg.

[10] : The augmented theory of successful intelligence. In R. J. Sternberg (Ed.), Cambridge handbook of intelligence (2nd ed. Vol. 2). *Cambridge University Press.* .2020;679-708

[11] : Adaptive intelligence: Surviving and thriving in a world of uncertainty. *Cambridge University Press* .2021;

[12] : What Is Wisdom? A Unified 6P Framework. *Review of General Psychology* .2021;25(2) : 10.1177/1089268020985509 134-151 10.1177/1089268020985509

# Response

**Published:** 2021-09-21

**Authors:** Stefan Arold

**Affiliations:** Biological and Environmental Science and Engineering Division, King Abdullah University of Science and Technology, Saudi Arabia

I thank Prof. Sternberg for his thoughtful comments and criticism. These comments add greatly to the discussion I was hoping to engage in, and I welcome that the F1000 format allows publishing them alongside the manuscript. Below, I have represented passages from the reviewer’s report in italics. My replies are below.

*[…] I believe the evidence supports the contention that human-type intelligence has many maladaptive aspects, but I am not convinced that the issue is evolutionary. Moreover, much depends on what one means by “human intelligence.” Perhaps the greatest problem with the article as it now stands is the definition of human intelligence. The definition in the article is “the intelligence that enables development of advanced technology-based societies like ours.” This definition of intelligence is nonstandard and appears to be idiosyncratic relatively to standard definitions in the literature on intelligence, which tend to emphasize ability to learn, to adapt to the environment, to reason, and to process information efficiently and effectively (see definitions in Sternberg, 2020a).*

 I agree that my definition of “HI” is non-standard, and acknowledge that this definition is “deliberately and necessarily […] very narrow and anthropocentric” in my manuscript. Indeed, this definition is specific for “HI”, formulated to fit the purpose of my manuscript. It should not be seen as an alternative definition for “general intelligence”.

 The main purpose of my manuscript is to ask why our particular intelligence “has never been paralleled by any other organism”. To position this discussion, I needed a definition of human intelligence that captures its unique attributes. However, many aspects of human intelligence (e.g. articulate communication, tool use, intentional cultivation of other species, intentional changes to the environment, culture and teaching) are also identified in other animals. In fact, our human intelligence appears to be a mix of cognitive traits that are already present in other animals. This observation is understandable because our brain is genetically and mechanistically not fundamentally different from the animal brains from which it derives. It is only the increased complexity and size of the human brain that enhance many of these traits. I also agree with Prof. Sternberg that intelligence is multidimensional and cannot be reduced to a single quantifying entity such as the IQ (e.g. Sternberg, 2019a). Hence, I chose the “development of technology-based societies” as the uniquely human hallmark of HI. Were we to discover life on another planet, then it would be this “technology” aspect of intelligence that we would take (rightly or wrongly) as an indicator for an alien species to be our equal in terms of intelligence.

 To clarify that my purpose-built definition of HI is not a general definition of (animal/human) intelligence I have added a sentence to the ‘Introduction’: “This definition is specific for “HI”, formulated to fit the purpose of my manuscript. It should not be seen as an alternative definition for “general intelligence”, and I now cite *Sternberg, 2020a.*

*The problem with the author’s definition is that, for most of human history, there was no serious technology, at least not in the sense discussed in the article.  If the goal of intelligence is to enable technology, then it has to be a recent one, not one that is evolutionarily-based. Technology as we know it today is too recent. Yet, the functions of intelligence actually do change over time (Greenfield, 2020) and are somewhat different as a function of culture and place (Sternberg, 2012). If technology is the goal, and so much of technology, as we now know, is destructive, it is no surprise that intelligence could be so dangerous, whether for evolutionary or other reasons*.

 In my manuscript, I am following the proposition of S.J. Gould that the human brain is an exaptation, not an adaptation (S.J. Gould, 1991; mentioned in my manuscript). Accordingly, advanced technology was not the evolutionary goal of HI­—just as powered bird flight was not the evolutionary goal of feathers, which have evolved many million years before birds took off from the ground (doi: 10.1016/j.tree.2019.04.018). Of course, a bird would not be able to fly without feathers, just as humans would not be able to produce technology without their brains. Yet, just as flapping or gliding with enlarged and specialised feathers may have increased the chances of survival in the ancestors of birds, the enlarged and specialised brains may have done the same for early homo species, for example by enhancing tool use or the control of fire. Intriguingly, many bird species coexist, and many species from other genera have developed highly sophisticated adaptations to allow powered flight without feathers. Conversely, only humans have developed ”serious” technology, and only one human species survives today. Given that even complex beneficial features such as powered flight could arise many times during the past ~200 million years, why has no other species developed cognitive capabilities that ultimately produce an advanced technology?

*The argument that human-type intelligence is maladaptive fits into an existing literature, which is largely not cited.  Consider some examples. First, there are notions of intelligence that are simply different from the conventional one. For example, Sternberg (2019a, 2019b, 2021), in speaking of “adaptive intelligence,” has argued that current human deployment of intelligence has become destructive not only to humans, but also to many other species. It is making the environment unsustainable for human life, a point with which the author apparently agrees. Second, there are broader notions of intelligence that suggest that the conventional view of intelligence is simply too narrow (Gardner, 2011; Sternberg, 2020b)—that there are multiple intelligences that do not fit conveniently into a single notion of general human intelligence. Third, there are theories of different kinds of intelligence that suggest that the conventional notion simply misses important aspects of human functioning, for example, emotional intelligence (e.g., Rivers et al., 2020), cultural intelligence (Ang et al., 2020), and practical intelligence (e.g., Hedlund, 2020). Fourth, there is the literature on wisdom, suggesting that humans need to apply their intelligence toward some kind of common good in order to thrive and create a sustainable future for themselves (e.g., Grossmann et al., 2020; Sternberg & Karami, 2021). Finally, dystopian novels, such as Brave New World and 1984, have shown how human intelligence can lead to catastrophe as well as to success.*

 In my manuscript, I conclude that the development of the features needed to create HI (in my definition) is not “difficult” enough (in terms of converging evolution) to justify that no other species has ever paralleled our cognition. As a possible explanation for this conundrum, I then speculate that HI is an intrinsically unfavourable trait, meaning that “the same mental and behavioural characteristics that are necessary for the development of HI might become increasingly unfavourable as a species evolves towards HI, creating a negative feedback loop”.

 In my manuscript, I based the current maladaptiveness of HI (in humans) on scientific data and predictions that state that our path is unsustainable and will lead to the “destruction of the resources onto which we critically depend”. Hence, I have only cited (some of) these sources. Evidence for the unsustainable nature of our trajectory is accumulating rapidly, and I have now included the IPCC Sixth Assessment Report to add a most recent reference.

 The points raised by Prof. Sternberg are of course relevant, and I thank him for bringing additional aspects into this discussion. Indeed, given that our intelligence is so highly “adaptive” that it allowed us to invade and populate all continents, it is puzzling that our intelligence appears to be consciously maladaptive in the long run with respect to the environment that we have largely created ourselves. Homo sapiens is a great invasive species but lacks the wisdom to sustain itself, once there are no new territories to conquer? Is it the general nature of HI to destroy itself? This discussion extends beyond the focus of my manuscript (which is: are there intrinsic feedback loops that hamper the development of HI in other species?).

 Concerning the literature on the different types of intelligence, I refer to my first reply above that I hope justifies why I have chosen a specific ‘result-based’ definition of HI (not general intelligence) for the purpose of this manuscript.

 The two dystopian novels deal with the author’s imaginations for how future societies contain intellectual or moral aspects of human intelligence/behaviour. Destruction of the environment was not yet an issue. With this new imminent threat to humanity in plain view, what would Huxley’s brave new world have been?

*I think there is good evidence that human intelligence can be maladaptive. We scarcely need laboratory experiments when we look at how humans have handled crises such as global climate change, pollution, pandemics, and income disparity.  But are the mechanisms gravitating against human intelligence in animals other than humans evolutionary? That is much less clear.  No other species has, to our knowledge, come close to destroying the environment in the way humans have. There is no evidence that, because they started destroying the environment, evolution of human-like intelligence stopped. Moreover, for an evolutionary mechanism to take hold, the maladaptive trait would likely interfere with reproductive capability, but the people who destroy the Earth are mostly professionals, from politicians to corporate leaders, who are largely past reproductive age.  By the time they succeed in destroying the environment, they are older. Serious mental illness, cited in the article, can interfere with reproduction, but it seems to apply to relatively small proportions of the human population.*

 I agree with Prof. Sternberg’s comments and criticism above: I do not claim to have fully resolved the nature of the intrinsic negative feedback that I propose limits the development of HI in species. In fact, my main concern was to first acknowledge that “something” appears to be intrinsically limiting the development of HI in animals because producing the physiological framework for HI is not sufficiently “difficult” to justify that no other animal has ever reached it. As possible intrinsically limiting factors, I suggest the resulting fragilizing complexity (of organisms and the societies they form) and resource-depleting technology. I provide some illustrations of these concepts based on non-human animals (pre-HI, according to my definition) in my manuscript.

 To tentatively explain that HI did evolve in humans, despite these intrinsically limiting mechanisms, I hypothesise that the human brain, and hence its HI capability, has developed rapidly as an exaptation in a low-abundance species, and thus has managed to circumvent these intrinsically limiting mechanisms. Will the same mechanisms now be able to push back future human descendants into a pre-HI stage? As mentioned in my manuscript, there are interesting signs of “self-domestication”, and the relative brain volume of humans appears to have been shrinking in *H. sapiens*, when compared to earlier homo species (but this could also be a sign of optimising the brain’s efficiency). Air/water pollution and mental health problems are reducing fertility. But are these evolutionary mechanisms rapid enough to change our brain and nature in time for a non-catastrophic transition? Probably not, alas.

*Although I have some disagreements with the author, I agree that “Despite the development of psychological, moral, behavioural (‘self-domestication’), pharmacological and technological solutions, we are knowingly and consciously pursuing our unsustainable development that is rapidly destroying the resources upon which we critically depend. However, globally engaging in the actions required to change our current trajectory and achieve long-term sustainability appears contrary to human nature.”*

*Unless intelligence is deployed more adaptively, the world and the creatures in it are at serious risk (Sternberg, 2021). The question is whether we want to create a future for our progeny over many generations, or rather for many generations of viruses, bacteria, and, inevitably, the ever-present cockroaches.*

 Unfortunately, rather than changing our ways to favour our progeny, we seemingly prefer not to change and rather wait for a *deus ex machina*—with a *machina* being created by the same technology, and a *deus* with the same nature, that put us (and the rest of the planet) in this dire situation. A rational person would bet on the cockroach.

# Peer review: Reviewer response for version 2

**DOI:** 10.5256/f1000research.28862.r72866

**Published:** 2020-10-14

**Authors:** Atsushi Iriki, Rafael Bretas Vieira

**Affiliations:** 1 Laboratory for Symbolic Cognitive Development, RIKEN Center for Biosystems Dynamics Research, Kobe, Japan

The author's revision of the manuscript satisfactorily addresses all concerns raised. The responses open to the reader, together with the respective references added, provide a clear justification for the statements made and an insightful view on the process of thought that led to each statement.

Is the topic of the opinion article discussed accurately in the context of the current literature?

Yes

Are arguments sufficiently supported by evidence from the published literature?

Partly

Are all factual statements correct and adequately supported by citations?

Yes

Are the conclusions drawn balanced and justified on the basis of the presented arguments?

Yes

Reviewer Expertise:

Spatial navigation, neuro-electrophysiology, behavioral psychology, consciousness, bodily self-awareness.

We confirm that we have read this submission and believe that we have an appropriate level of expertise to confirm that it is of an acceptable scientific standard.

# Peer review: Reviewer response for version 1

**DOI:** 10.5256/f1000research.24305.r65187

**Published:** 2020-07-03

**Authors:** Atsushi Iriki, Rafael Bretas Vieira

**Affiliations:** 1 Laboratory for Symbolic Cognitive Development, RIKEN Center for Biosystems Dynamics Research, Kobe, Japan

The manuscript discusses the uniqueness of human intelligence (HI) despite its apparent evolutionary advantages. Although the theme has been tirelessly revisited by many authors, a new theory is offered, that higher intelligence is disadvantageous and kept from developing through negative feedback. The author briefly goes through the main molecular and ecological arguments for the appearance and advantages of high intelligence, showing that those are not especially uncommon or slow in evolutionary history. Therefore, none could fully justify the evolution of HI, suggesting it to be generally an unfavorable trait.

 The theory is well constructed and the references support the hypothesis. Despite that, there are a few points in the manuscript that warrant attention:

**Page 3 - "Brain size, structure, and complexity"**

 The author affirms that " *In terms of genetic modifications, the development of the human brain therefore appears to be based on extending already existing features through gradual and common mechanisms, rather than being the result of a developmental quantum leap" (page 3, para 5, line 33)*. The example given a few lines before, of Pax6, as well as other well known genes, such as the human-specific ARHGAP11B, despite being relatively small changes, result in significant leaps in brain development. The later example of octopuses also seems to indicate a sudden change, from the moment molluscs lost their shells, which still supports the paragraph conclusion that the genetic mechanisms that allow intelligence are available to other species.

**Page 5 - "Language and intelligence"**

 In general, this session is not clear about language being a requirement for the development of HI, a mere consequence of it, or something in between, with some contradictions. For example *"It is therefore not the capacity for an articulate vocalization, but only a predisposition for it, that is essential to the evolution of HI" (page 5, para 5, line 11)* and *"current evidence suggests that the evolution of a HI-supporting language is not a feature strictly limited to humans or the human brain anatomy or speech production" (page 5, para 7, line 6)*. The conclusion is especially confusing when declaring "HI-supporting language" while the manuscript appears to state that language is not a prerequisite for HI.

 About the passage:

*"Let us now consider language syntax and acquisition with respect to human uniqueness. It has indeed been proposed that such properties of the human language are neither specific to language nor to humans (47,48)" (page 5, para 6, line 1)*. The references cited (47, 48) indicate that there may be a human-only syntax, either through universal grammar or simply an advanced form of syntax allowed by the human FLB.

**Page 7 - "Conclusion and outlook"**

 Despite mentioning the possible negative feedback from molecular complexity, I couldn't find any examination of it in the main text. For reference: *"the main barrier towards HI is not only the development of the required physical assets, but also that negative feedback from social, behavioural and molecular complexity, as well as negative environmental feedback make the development of HI increasingly unfavourable" (page 7, para 4, line 11)*.

 I wonder if there is a link between HI and sexual selection. Although intelligence as a simple fitness indicator would be equally accessible to other animals as well, cultural and behavioral products could also have influenced mating preferences.

 Finally, the text mentions the possible negative feedback of niche construction: page 7, *"there might be a different type of negative feedback loop intrinsic to HI—HI might simply allow a species to become so successful in exploiting food resources that these resources become exhausted"  (page 7, para 3, line 1)* and again on the conclusion *"we are knowingly and consciously pursuing our unsustainable development that is rapidly destroying the resources upon which we critically depend" (page 7, para 6, line 6)*. Despite that, HI also generates powerful positive feedback with niche construction, in which the environmental changes produced by HI favor HI itself.

Is the topic of the opinion article discussed accurately in the context of the current literature?

Yes

Are arguments sufficiently supported by evidence from the published literature?

Partly

Are all factual statements correct and adequately supported by citations?

Yes

Are the conclusions drawn balanced and justified on the basis of the presented arguments?

Yes

Reviewer Expertise:

Spatial navigation, neuro-electrophysiology, behavioral psychology, consciousness, bodily self-awareness.

We confirm that we have read this submission and believe that we have an appropriate level of expertise to confirm that it is of an acceptable scientific standard, however we have significant reservations, as outlined above.

# Response

**Published:** 2020-08-14

**Authors:** Stefan Arold

**Affiliations:** Biological and Environmental Science and Engineering Division, King Abdullah University of Science and Technology, Saudi Arabia

I thank Dr. Atsushi and Dr. Vieira for their time and efforts to critically evaluate my manuscript, and for their very constructive feedback. In the following I provide a point-by-point reply to their comments, outlining also the changes I have made to the manuscript. I hope that my explanations (given below, which are accessible to readers) and manuscript changes respond to the reviewer’s comments in a satisfactory manner. Should issues remain, I’d be happy to further discuss and integrate these.

**Reply to comment on Page 3 –“ Brain size, structure and complexity”**

The reviewers refer to my statement that " *In terms of genetic modifications, the development of the human brain, therefore, appears to be based on extending already existing features through gradual and common mechanisms, rather than being the result of a developmental quantum leap" (page 3, para 5, line 33)*. They note that the “ *relatively small changes [in genes] result in significant leaps in brain development*.”

I thank the reviewers for this comment that identifies a poor formulation in my essay. Indeed, I argue (in agreement with the reviewers) that the apparently big anatomical and cognitive changes were caused by only relatively small genetic changes. Consequently, I have corrected the wording into: “ *rather than being the result of a **genetic** quantum leap.”*

**Reply to comment on Page 5 - "Language and intelligence"**

The reviewers note that “ *this session is not clear about language being a requirement for the development of HI, a mere consequence of it, or something in between, with some contradictions*”, and they give specific examples for this contradiction.

                 My position is that ‘full-blown’ HI needs a powerful communication system; however, Australopithecines and very early homo forms did certainly not have the language necessary to produce modern legislation, Shakespearean theatre or comprehensive gene ontologies. Hence, although a powerful communication system is a requirement for HI, it can (and probably will) be developed while a species develops HI. I agree that some of my formulations lacked precision, and I have now amended the text to enhance the clarity of this message:

“ *we then have to ask whether the **development** of HI-supporting language would be possible for other species*”.

*“It was therefore not the **pre-existence** of an articulate vocalisation, but only **a physiological potential** for it, that **was sufficient for allowing** the evolution of HI **in humans**”.*

“ *Thus, current evidence suggests that the **capacity for developing a** HI-supporting **system of communication** is not a feature strictly limited to humans or the human brain anatomy or speech production.*”

I thank Dr. Atsushi and Dr. Vieira for pointing out the mismatch between references and statement in the passage “let us now consider language syntax […]47,48”. Indeed, these references should not have been mentioned at this position, because the references for this statement follow in the ensuing discussion (references 50-57). Therefore, in the revised version, I have deleted the references 47,48 at this position (they are still used in the preceding passage) and put a colon instead of the full-stop for additional clarity.

**Reply to comment on Page 7 - "Conclusion and outlook"**

The reviewers rightly ask: “ *Despite mentioning the possible negative feedback from molecular complexity, I couldn't find any examination of it in the main text*.”. Indeed, in the initial version, ‘molecular complexity’ refers to the extension of protein-level amino-acid repeats that are linked to both, increased cognition, but also increased psychopathology. I gave the protein huntingtin as an example. However, I agree that such repeat extensions (which are frequently linked to cognition and neurological pathologies) are not adequately described as ‘molecular complexity’. Moreover, the genetics of mental illness are only emerging very slowly, with different studies often reaching different conclusions (for a summary, please see M. Marshall, Nature 581, 19-21 (2020) *doi: 10.1038/d41586-020-00922-8*). Hence, it is also premature for general correlations between complexity and psychopathology on a molecular level.

However, over the last decade, a good consensus has been reached that mental disorders can involve altered connectivity in our highly complex brains (e.g. Fornito and Harrison, Front. Psychiatry, 27 July 2012 https://doi.org/10.3389/fpsyt.2012.00072). My manuscript already refers to it in “ *HI as an intrinsically unfavourable trait*”, p7, in the two sentences following the mentioning of huntingtin (“[…] *the genes regulating synaptogenesis and neuronal circuit formation have been associated with an increased risk of mental illnesses. *[…] *brain networks involved in HI-required cognitive skills, such as language and complex social behaviour, have less compensatory mechanisms, and are hence less robust, than more ancient biological functions*”. Hence, to respond to the reviewers’ comment, I have changed ‘ *molecular complexity*’ into ‘ *neuroanatomical complexity*’ in this sentence.

The reviewers also ‘ *wonder if there is a link between HI and sexual selection*’. This is an interesting suggestion, and my thoughts on it are as follows: Interspecies variations in HI would not change the outside appearance and appeal of one individual with respect to a rival. However, above-average HI might indirectly influence mating preferences, for example through resulting in a higher in-group social status, better food sources (and hence physical fitness), and, at a more advanced stage, more attractive cultural/ornamental items. However, given the wide-spread occurrence of sexual selection across animal species, I don’t see how sexual selection would uniquely favour HI in homo, but not in any other species. Hence, while sexual selection might have an influence, this influence would not be specific enough to humans to justify that HI has only emerged in our lineage.

In their final comment, the reviewers refer to my discussion on the possible negative feedback of niche construction. In particular, they refer to my discussion of data showing the unsustainable nature of HI in its current ‘implementation’ in modern humans, ultimately making HI a maladaptive feature [“ *HI might […] allow a species to become so successful in exploiting food resources that these resources become exhausted"; and “we are knowingly and consciously pursuing our unsustainable development that is rapidly destroying the resources upon which we critically depend"*]. The reviewers suggest that “ *despite that, HI also generates powerful positive feedback with niche construction, in which the environmental changes produced by HI favour HI itself*.”

In response, I argue that both statements do not contradict each other. Rather, they consider different effects resulting in this feedback mechanism.

Niche construction theory states that acquired features that allow a species to favourably transform its environment can be selected for during evolution. If a species can use its intelligence to ‘consciously’ and favourably alter its environment, then this would certainly result in positive feedback for the development of HI. The dam-building beaver is an obvious example for ‘intelligent’ niche construction (as opposed to, say, cyanobacteria or earthworms). Hence, I agree with the reviewers and existing literature (e.g. doi:10.1098/rstb.2010.0306) that ‘conscient’ niche construction can boost the development of HI. However, two aspects are important in the context of my essay:

          Firstly, nothing indicates that the positive feedback between HI and the development of ‘conscient’ niche construction is limited to humans. Hence, niche construction would not explain the main focus of my manuscript, namely the uniqueness of HI in humans. To take into account the reviewer’s comments, and to directly clarify this point, I have added the general aspect of niche construction in my revised manuscript (see: Lack of suitable environment?: “ *Additionally, if the competitive advantage and driving force of HI development lies in overcoming dependence of the environment* *^14^* *, or enables favourable niche construction, then this advantage would profit many species, and hence is not a limiting factor*. “).

          Secondly, while niche construction may support the development of increased cognition, this synergy will become overall maladaptive if the changes to the environment deplete the food resources. As a non-human example, I have mentioned the study on island populations of long-tailed macaques. Obviously, through HI, humans were able to consciously alter their environment more than any other animal (cyanobacteria might have altered our planet even more than humans, but not ‘consciously’). However, as I note in my manuscript, available studies overwhelmingly conclude that these HI-enabled environmental changes are unsustainable because they are *“rapidly destroying the resources upon which we critically depend*”. Hence, the environment-transforming capacity of HI is now critically endangering the survival of our species—and this is the ‘increasingly negative side effects’ of our HI that I refer to in the ‘Conclusion and Outlook’ section.
